# Stage‐specific characterization of “early‐onset colorectal cancer”: Localized and synchronous metastatic disease

**DOI:** 10.1002/ijc.35336

**Published:** 2025-01-30

**Authors:** Erman Akkus, Beliz Bahar Karaoğlan, Mehmet Kayaalp, Utkucan Turmuş, Cihangir Akyol, Güngör Utkan

**Affiliations:** ^1^ Department of Medical Oncology Ankara University Faculty of Medicine Ankara Türkiye; ^2^ Ankara University Cancer Research Institute Ankara Türkiye; ^3^ Department of Internal Medicine Ankara University Faculty of Medicine Ankara Türkiye; ^4^ Department of Surgery Ankara University Faculty of Medicine Ankara Türkiye

**Keywords:** colorectal cancer, early onset, localized, *RAS*, synchronous metastatic

## Abstract

Early‐onset colorectal cancer (EOCRC) is an alarming entity worldwide. Yet, stage‐specific characteristics and prognosis in localized and synchronous metastatic EOCRC are not well‐defined. Two cohorts of CRC patients (localized and synchronous metastatic) were evaluated, defining EOCRC as the diagnosis <50 years old. Five hundred sixty‐eight patients were included (*n* = 432 localized, 14.4% [*n* = 62] EOCRC and *n* = 136 synchronous metastatic, 20.6% [*n* = 28] EOCRC). 93.5% of localized and 96.5% of synchronous metastatic EOCRC patients were symptomatic at diagnosis. Among localized patients, female gender (58.1% vs. 40%, *p* = .008), perineural invasion (41.9% vs. 24.9%, *p* = .005), folinic acid, 5‐fluorouracil, and oxaliplatin chemotherapy (45.2% vs. 25.2%, *p* = .003), and perioperative chemotherapy cycles (9.21 [± 3.10] vs. 7.98 [± 2.92], *p* = .006) were higher in EOCRC compared with ≥50‐year. Median recurrence‐free survival (RFS) and overall survival were not reached in either group (*p* = .234 and *p* = .831). Only *RAS* mutant status was associated with RFS (Hazard ratio: 7.09 [95% confidence interval (CI): 1.87–26.76], *p* < .001) in EOCRC. Among synchronous metastatic patients, urgent surgery (32.1% vs. 11.1%, *p* = .014) and local treatments (39.3% vs. 20.4%, *p* = .037) were more frequent in EOCRC. Median progression‐free survival and overall survival in the EOCRC and ≥50 years were 8.07 months (95% CI: 5.03–12.97) vs. 10.03 months (95% CI, 8.40–13.10) (*p* = .450) and 18.57 months (95% CI, 13.33–43.03) vs. 19.83 months (95% CI, 16.07–27.30) (*p* = .833), respectively. Synchronous metastatic EOCRC more frequently underwent urgent surgery (32.1% vs. 8%, *p* = .008) and had *RAS* mutation (43.5% vs. 16.7%, *p* = .032) than localized EOCRC. This study suggests that localized and synchronous metastatic EOCRC patients may have different characteristics than average onset, without survival differences. Implementation of stage‐specific characteristics into daily practice is necessary for decision‐making processes in these young patients.

## What's New?

The incidence of early‐onset colorectal cancer (EOCRC) has been raising, and stage‐specific characteristics and prognosis need to be clarified. In this study, more than 90% of EOCRC cases presented with symptoms. Among patients with localized cancer, female gender, perineural invasion, and intense treatment were more frequent in early than in average‐onset colorectal cancer. Among patients with synchronous metastatic cancer, urgent surgery and local treatments were more frequent in early‐onset colorectal cancer. Patients with localized and synchronous metastatic EOCRC showed different characteristics than patients with average onset, without survival differences. *RAS* mutant status emerged as a potential prognostic factor in EOCRC.

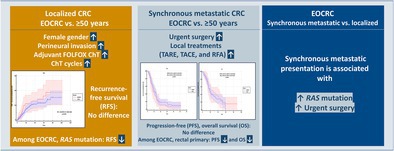

## 1 INTRODUCTION

Colorectal cancer (CRC) is the third most common cancer in males, the second in females, and the third most common cause of cancer mortality in both genders, despite varying incidence and mortality among regions.^1^ Although age is a risk factor for CRC, the incidence is decreasing in older adults; however, there is a steep increase in young‐ and middle‐aged adults. The incidence in individuals younger than 50 years is increasing by about 0.5% and 1.6% per year for colon cancer and 2% per year for rectal cancer.^2^ The proportion of newly diagnosed colon and rectal cancer at a young age has increased from 11% to 20% for colon cancer and from 15% to 28% for rectal cancer, respectively.^2^

Early‐onset colorectal cancer (EOCRC) is, thus, defined as CRC diagnosed before the age of 50 years.^3^ Most cases are sporadic and appear to be influenced by environmental risk factors, such as obesity and diet with poor quality.^2^, ^3^, ^4^ EOCRC patients are more likely to be female, more symptomatic, and more often diagnosed in advanced stages.^2^, ^5^ The data on prognosis of EOCRC patients are conflicting possibly due to the presence of factors such as aggressive tumor phenotype, potential overtreatment, better performance scores, and fewer comorbidities, all influencing prognosis.^6^ However, there is no investigation of stage‐specific characteristics, risk, and prognostic factor differences in localized and synchronous metastatic EOCRC. Understanding the stage‐specific characteristics of EOCRC is crucial for developing strategies for the screening, diagnosing, and management of the disease in young patients. Therefore, in this study, we aimed to investigate the stage‐specific characteristics and prognosis of patients with localized and synchronous metastatic EOCRC.

## 2 MATERIALS AND METHODS

### 2.1 Patient cohorts and study design

Two retrospective cohorts of patients with CRC were evaluated. Ankara University Faculty of Medicine Localized CRC Cohort (AUTF‐NMKRK) included 432 patients with localized CRC at the diagnosis between 2015 and 2024. Ankara University Faculty of Medicine Synchronous Metastatic CRC Cohort (AUTF‐DMKRK) included 136 patients with synchronous metastatic CRC between 2019 and 2023.

The data of the cohorts included demographic, surgical, pathological, laboratory, imaging, and clinical data. All data were retrieved from the Avicenna Hospital Data Management System to be recorded in the in‐house registry of our institute for the cohorts. Responsible physicians update data in every inpatient or outpatient visit of an individual patient. The cut‐off date for data collection was February 2024 for the study.

For patients with localized CRC, age, gender, comorbidities (diabetes, hypertension, coronary artery disease [CAD]), primary tumor location, presence of urgent surgery, T and N stages, and stage grouping (according to the Union for International Cancer Control TNM Classification of Malignant Tumours, 8th Edition),^7^ histopathological features (grade, mucinous component, lymphovascular invasion, perineural invasion [PNI], tumor deposits, budding), microsatellite instability status (MSI), *RAS‐BRAF* mutational status, ABO and Rh blood groups, perioperative treatments, and recurrence and survival data were included. For patients with synchronous metastatic CRC, age, gender, comorbidities (diabetes, hypertension, CAD), smoking history, primary tumor location, metastatic sites, plasma carcinoembryonic antigen, and carbohydrate antigen 19–9 levels at the diagnosis, presence of urgent surgery, MSI, *RAS‐BRAF* mutational status, ABO and Rh blood groups, systemic and local treatments, progression, and survival data were included.

*RAS* mutational status was analyzed by targeted sequencing and included exons 2, 3, and 4 of *KRAS* and *NRAS* genes. *BRAF* mutational status was analyzed by targeted sequencing and included the exon 15 codon 600 (*BRAF* V600E).

### 2.2 Outcomes

EOCRC was defined as CRC diagnosed before the age of 50 years (<50) for both localized and synchronous metastatic CRC cohorts. For localized CRC; demographic, clinical, and treatment characteristics were compared between EOCRC and ≥50‐year groups. Recurrence‐free survival (RFS) and overall survival (OS) of localized EOCRC were presented. Recurrence risk was compared between EOCRC and ≥50‐year groups. Additionally, the prognostic factors associated with recurrence in patients with localized EOCRC were analyzed. For synchronous metastatic CRC, demographic, clinical, and treatment characteristics, progression‐free survival (PFS), and OS were compared between EOCRC and ≥50‐year groups. Prognostic factors associated with PFS and OS in patients with synchronous metastatic EOCRC were analyzed. Finally, demographic and clinical characteristics were compared between localized and synchronous metastatic EOCRC patients.

### 2.3 Statistical analysis

Continuous variables were given as median (minimum [min]–maximum [max]), and categorical variables were presented as the percentage. Univariable comparisons were performed using chi‐square, Fisher exact, Student's *t*, Mann–Whitney *U*‐tests, and Cox regression, where needed. The statistically significant variables in the univariable analysis were included in the multivariable analysis. Multivariable analyses were performed by logistic regression and Cox regression analyses. Survivals were estimated by the Kaplan–Meier method and compared by log‐rank test. Sensitivity analyses were performed for the variables that had missing data.^8^ Multiple imputation technique using regression method was utilized.^9^ Pooled *p*‐values of sensitivity analyses were presented. All *p*‐values were based on a two‐tailed test of significance (*p* = .05). All the statistical analyses were conducted using the software SPSS version 26 (SPSS Inc., USA) and the MedCalc® Statistical Software version 22.026 (MedCalc Software Ltd., Ostend, Belgium).

## 3 RESULTS

### 3.1 Localized EOCRC

Among 432 patients with localized CRC, 14.4% (*n* = 62) were classified as EOCRC (Table 1). The median age of EOCRC patients was 46 (20–49), and 58.1% were female. 33.8% (*n* = 21) of the cases had right‐sided tumors. 24.2% of patients had pT4 tumors, and 50% of patients had lymph node metastasis, respectively. Additionally, 45.2% of tumors showed lymphovascular invasion and 41.9% of tumors showed PNI. Notably, 12.9% (*n* = 8) of patients had tumors with MSI‐H. Urgent surgery due to obstruction was needed for 8.1% (*n* = 5) of the localized EOCRC patients.

**TABLE 1:** Characteristics^+^ of patients with localized colorectal cancer (CRC) (^+^Multivariable logistic regression and Cox regression analyses were presented in Tables S1–S5).

Variable	All patients	EOCRC	≥50‐year CRC	*p*‐value
	*n* = 432	*n* = 62 (14.4%)	*n* = 370 (85.6%)	
Age, median (min–max)	63 (20–90)	46 (20–49)	65 (50–90)	.**000**
Gender, *n* (%)				
Male	248 (57.4)	26 (41.9)	222 (60)	.**008**
Female	184 (42.6)	36 (58.1)	148 (40)	
Diabetes, *n* (%)	81 (18.8)	5 (8)	76 (20.5)	.**019**
Hypertension, *n* (%)	110 (25.5)	3 (4.8)	107 (28.9)	.**000**
CAD, *n* (%)	58 (13.4)	1 (1.6)	57 (15.4)	.**003**
Primary tumor location, *n* (%)				
Right	127 (29.4)	16 (25.8)	111 (30)	.274
Transverse	19 (4.4)	5 (8)	14 (3.8)	
Left	169 (39.1)	21 (33.9)	148 (40)	
Rectum	115 (26.6)	20 (32.3)	95 (25.7)	
Urgent surgery, *n* (%)				
No	408 (94.4)	57 (91.9)	351 (94.9)	.212
Obstruction	19 (4.4)	5 (8.1)	14 (3.8)	
Perforation	5 (1.2)	0 (0)	5 (1.3)	
T stage, *n* (%)				
1	10 (2.3)	0 (0)	10 (2.7)	.237
2	33 (7.6)	3 (4.8)	30 (8.1)	
3	313 (72.5)	44 (71)	269 (72.7)	
4	76 (17.6)	15 (24.2)	61 (16.5)	
N stage, *n* (%)				
0	238 (55.1)	31 (50)	207 (55.9)	.835
1a‐b	115 (26.6)	18 (29)	97 (26.2)	
1c	33 (7.6)	5 (8)	28 (7.6)	
2	46 (10.6)	8 (13)	38 (10.3)	
Stage grouping (UICC, 8th)				
Stage I	28 (6.5)	2 (3.2)	26 (7)	.438
Stage II	210 (48.6)	29 (46.8)	181 (48.9)	
Stage III	194 (44.9)	31 (50)	163 (44.1)	
Tumor deposit, *n* (%)	56 (13)	8 (12.9)	48 (13)	.988
Tumor grade, *n* (%)				
1	23 (5.3)	3 (4.8)	20 (5.4)	.971/.779^a^
2	335 (77.5)	45 (72.6)	290 (78.4)	
3	40 (9.3)	6 (9.7)	34 (9.2)	
UK	34 (7.9)	8 (12.9)	26 (7)	
Mucinous component, *n* (%)	80 (18.5)	15 (24.2)	65 (17.6)	.214
LVI, *n* (%)	166 (38.4)	28 (45.2)	138 (37.3)	.239
PNI, *n* (%)	118 (27.3)	26 (41.9)	92 (24.9)	.**005**
Budding, *n* (%)	63 (14.6)	7 (11.3)	56 (15.1)	.427
MSI‐H, *n* (%)	37 (8.6)	8 (12.9)	29 (7.8)	.234/.682^a^
*KRAS or NRAS*, *n* (%)				
WT	177 (41)	26 (41.9)	151 (40.8)	.552/.988^a^
Mutant	25 (5.8)	5 (8.1)	20 (5.4)	
UK	230 (53.2)	31 (50)	299 (80.8)	
*BRAF V600E*, *n* (%)				
WT	196 (45.4)	29 (46.7)	4 (1)	0.558/1^a^
Mutant	5 (1.2)	1 (1.6)	167 (45.1)	
UK	231 (53.5)	32 (51.7)	299 (80.8)	
ABO group, *n* (%)				
AB	28 (6.5)	6 (9.7)	22 (5.9)	.413/.299^a^
A	194 (44.9)	31 (50)	163 (44)	
B	61 (14.1)	6 (9.7)	55 (14.9)	
O	134 (31)	17 (27.4)	117 (31.6)	
UK	15 (3.5)	2 (3.2)	13 (3.6)	
Rh, *n* (%)				
Positive	374 (86.6)	50 (80.6)	324 (87.6)	.080/.298^a^
Negative	43 (10)	10 (16.1)	33 (8.9)	
UK	15 (3.5)	2 (3.2)	13 (3.6)	

*Note*: Statistically significant *p* values (based on a two‐tailed test of significance, *p* = .05) were written bold.

Abbreviations: CAD, coronary artery disease; EOCRC, early‐onset colorectal cancer; LVI, lymphovascular invasion; MSI‐H, microsatellite instability‐high; PNI, perineural invasion; UK, unknown; WT, wild type.

^a^ Sensitivity analyses were performed for the missing data. Pooled *p*‐values of multiple imputations in sensitivity analysis were presented.

Among the localized CRC cohort, the EOCRC group had a higher proportion of females compared to the ≥50‐year group (58.1% vs. 40%, *p* = .008). Diabetes, hypertension, and CAD were more commonly seen in the ≥50‐year group (20.5% vs. 8%, *p* = .019; 28.9% vs. 4.8%, *p* = .000 and 15.4% vs. 1.6%, *p* = .003, respectively). PNI was more common among the EOCRC group than the ≥50‐year group (41.9% vs. 24.9%, *p* = .005) (Table 1). Female gender (OR: 2.17, 95% confidence interval [CI]: 1.24–3.77, *p* = .006) and presence of PNI (OR: 2.28, 95% CI: 1.30–4.02, *p* = .008) remained statistically significant for EOCRC when assessed in multivariable binary logistic analysis (Tables S1 and S2).

Seventy‐nine percent of the localized EOCRC patients received adjuvant chemotherapy. Compared with the ≥50‐year group, EOCRC patients more frequently received folinic acid, 5‐fluorouracil, and oxaliplatin chemotherapy (45.2% vs. 25.2%, *p* = .003), while oxaliplatin, capecitabine, and capecitabine regimens were more commonly administered in the ≥50‐year group. Perioperative chemotherapy cycles were significantly higher in the EOCRC group compared with the ≥50‐year group (9.21 (± 3.10) vs. 7.98 (± 2.92), *p* = .006). Patients with rectal cancer received neoadjuvant radiotherapy and chemotherapy (Table 2).

**TABLE 2:** Treatment characteristics^+^ of localized colorectal cancer (CRC) (^+^Multivariable logistic regression and Cox regression analyses were presented in Tables S1–S5).

Variable	All patients	EOCRC	≥50‐year CRC	*p*‐Value
	*n* = 432	*n* = 62, 14.4%	*n* = 370, 85.6%	
Perioperative treatment, *n* (%)	347 (80.3)	53 (85.5)	294 (79.6)	.269
Adjuvant chemotherapy, *n* (%)				
No	103 (23.9)	13 (21)	90 (24.3)	.**003**
XELOX	141 (32.6)	16 (25.8)	125 (33.8)	
FOLFOX	114 (26.4)	28 (45.2)	86 (23.2)	
Capecitabine	74 (17.1)	5 (8)	69 (18.6)	
Neoadjuvant radiotherapy, *n* (%)				
Not received	347 (80.3)	44 (71)	303 (81.9)	.152
Short course (5 × 5)	18 (4.2)	4 (6.4)	14 (3.8)	
Long course (CRT)	67 (15.5)	14 (22.6)	53 (14.3)	
Neoadjuvant chemotherapy, *n* (%)				
No	395 (91.5)	54 (87.1)	341 (92.2)	.422
XELOX	32 (7.4)	7 (11.3)	25 (6.8)	
FOLFOX	5 (1.2)	1 (1.6)	4 (1.1)	
Perioperative chemotherapy cycle				
Median (min–max)	8 (0–12)	8 (0–12)	8 (0–12)	.**005**
Mean (SD)	8.17 (± 2.98)	9.21 (± 3.10)	7.98 (± 2.92)	.**006**

*Note*: Statistically significant *p* values (based on a two‐tailed test of significance, *p* = .05) were written bold.

Abbreviations: CRT, chemoradiotherapy; EOCRC, early‐onset colorectal cancer; FOLFOX, folinic acid, 5‐fluorouracil, and oxaliplatin; SD, standard deviation; XELOX, oxaliplatin and capecitabine.

**FIGURE 1:**
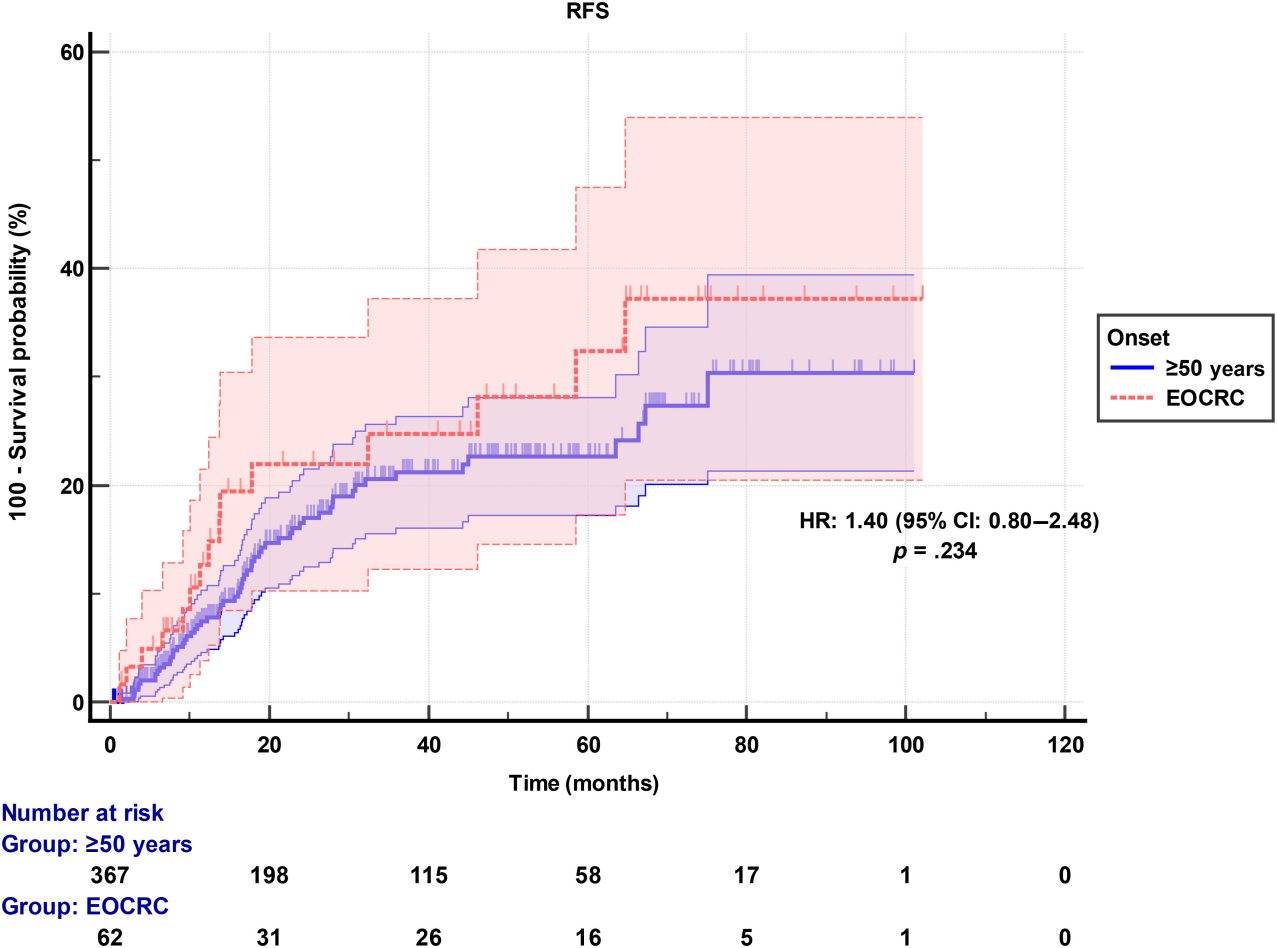
Recurrence‐free survival of early‐onset colorectal cancer (EOCRC) and ≥50‐year‐old localized colorectal cancer patients.

The median follow‐up time for the entire population was 28.6 months (1.3–102.1). During the follow‐up, recurrence occurred in 17.6% (*n* = 76) of the patients. The recurrence rate was 24.2% (*n* = 15) in the EOCRC group in a median follow‐up time of 37.9 months (1.4–102.1), whereas 16.5% (*n* = 61) in the ≥50‐year group in a median follow‐up time of 28.1 months (1.3–101.0). Median RFS and OS were “Not reached” in both groups with no statistical difference (*p* = .234 and *p* = .831, respectively) (Figure 1).

Demographic, clinical, and treatment characteristics were analyzed to investigate their association with RFS in localized EOCRC patients. Only *RAS* mutant status was associated with RFS in the univariable analysis (Hazard ratio [HR]: 7.09 [95% CI: 1.87–26.76], *p* < .001) (Table S3). Sensitivity analyses revealed consistent results (HR: 15.06 [95% CI: 3.35–67.73], *p* = .001) (Table S3). When the variables with *p* < .20 included the multivariable analysis, the presence of *RAS* mutation (HR: 14.25 [95% CI: 2.61–77.61], *p* = .002) and N stage (N1c vs. N0, HR: 51.92 [95% CI: 4.36–618.08], *p* = .002) were associated with RFS (Table S4). After adjusting additionally by age, gender, comorbidities, and tumor location, *RAS* mutation status remained associated with RFS (Table S5). In the sensitivity analyses for multivariable assessment, only *RAS* mutant status remained significant (HR: 14.83 [95% CI: 3.00–73.15], *p* = .001) (Table S4).

### 3.2 Synchronous metastatic EOCRC

Among 136 patients with synchronous metastatic CRC, 20.6% (*n* = 28) were EOCRC (Table 3). Median age was 45 (30–49), and 60.7% (*n* = 17) of the patients were male. 46.4% (*n* = 11) had rectal cancer. 32.4% (*n* = 9) of patients underwent urgent surgery. Liver metastases were present in 92.9% (*n* = 26) of the patients.

**TABLE 3:** Characteristics^+^ of patients with synchronous metastatic colorectal cancer (CRC) (^+^Cox regression analyses for progression‐free survival and overall survival were presented in Table S6).

Variable	All patients	EOCRC	≥50‐year CRC	*p*‐Value
	*n* = 136	*n* = 28 (%20.6)	*n* = 108 (%79.4)	
Age, median (min–max)	60 (30–80)	45 (30–49)	63 (50–80)	.**000**
Gender, *n* (%)				
Male	84 (61.8)	17 (60.7)	67 (62)	.898
Female	52 (38.2)	11 (39.3)	41 (38)	
Diabetes, *n* (%)	26 (19.1)	1 (3.6)	25 (23.1)	.**019**
Hypertension, *n* (%)	43 (31.6)	3 (10.7)	40 (37)	.**008**
CAD, *n* (%)	13 (9.6)	0 (0)	13 (12)	.070
Smoking history, *n* (%)	70 (51.5)	15 (53.6)	55 (50.9)	.803
Location, *n* (%)				
Colon	79 (58.1)	15 (53.6)	64 (59.3)	.613
Rectum	57 (41.9)	13 (46.4)	44 (40.7)	
Urgent surgery, *n* (%)	21 (15.4)	9 (32.1)	12 (11.1)	.**014**
MSI, *n* (%)				
MSI‐H	3 (2.2)	2 (7.1)	1 (1)	.132/1^a^
MSS	88 (64.7)	19 (67.9)	69 (63.9)	
UK	45 (33.1)	7 (25)	38 (35.1)	
*KRAS or NRAS*, *n* (%)				
WT	45 (33.1)	13 (46.4)	32 (29.6)	.173/.579^a^
Mutant	57 (41.9)	10 (35.7)	47 (43.5)	
UK	34 (25)	5 (17.9)	28 (25.9)	
*BRAF V600E*, *n* (%)				
WT	79 (58.1)	20 (71.4)	59 (54.6)	.316/.961^a^
Mutant	3 (2.2)	0 (0)	3 (2.8)	
UK	54 (39.7)	8 (28.6)	46 (42.6)	
Liver metastasis, *n* (%)	120 (88.2)	26 (92.9)	94 (87)	.524
Peritoneal metastasis, *n* (%)	18 (13.2)	1 (3.6)	17 (15.7)	.121
Lung metastasis, *n* (%)	35 (25.7)	8 (28.6)	27 (25)	.700
Bone metastasis, *n* (%)	6 (4.4)	0 (0)	6 (5.6)	.343
CEA, ng/mL, median (min–max)	38.75 (0.93–17,796)	52.20 (0.93–1065)	38.67 (0.94–17,796)	.677
Ca19‐9, U/mL median (min–max)	93.50 (0.80–19,300)	39.60 (0.80–19,300)	99.50 (0.80–12,443)	.282
ABO group, *n* (%)				
AB	10 (7.4)	2 (7.1)	8 (7.4)	.480/.280^a^
A	53 (39)	10 (35.7)	43 (39.8)	
B	20 (14.7)	4 (14.3)	16 (14.8)	
O	30 (22.1)	10 (35.7)	20 (18.6)	
UK	23 (16.9)	2 (7.1)	21 (19.4)	
Rh, *n* (%)				
Positive	99 (72.8)	24 (85.8)	75 (69.4)	.516/.767^a^
Negative	14 (10.3)	2 (7.1)	12 (11.2)	
UK	23 (16.9)	2 (7.1)	21 (19.4)	
First‐line treatment, *n* (%)				
5FU‐OX doublet	73 (53.7)	12 (42.8)	61 (56.5)	.290
5FU‐OX doublet‐bevacizumab	30 (22.1)	10 (35.7)	20 (18.6)	
5FU‐OX doublet‐anti‐EGFR	23 (16.9)	3 (10.7)	20 (18.6)	
Only 5FU	1 (0.7)	1 (3.6)	0	
5FU‐IRI doublet	1 (0.7)	0 (0)	1 (<1)	
5FU‐IRI doublet‐bevacizumab	1 (0.7)	0 (0)	1 (<1)	
5FU‐IRI doublet‐anti‐EGFR	2 (1.5)	1 (3.6)	1 (<1)	
Triplet	1 (0.7)	0 (0)	1 (<1)	
Triplet‐bevacizumab	3 (2.2)	1 (3.6)	2 (1.8)	
Triplet‐anti‐EGFR	1 (0.7)	0 (0)	1 (<1)	
Local treatment, *n* (%)				
Surgery	24 (17.6)	3 (10.7)	21 (19.4)	.406
TARE‐TACE‐RFA	33 (24.3)	11 (39.3)	22 (20.4)	.**037**

*Note*: Statistically significant *p* values (based on a two‐tailed test of significance, *p* = .05) were written bold.

Abbreviations: 5FU, 5‐fluorouracil; Ca19‐9, carbohydrate antigen 19–9; CAD, coronary artery disease; CEA, carcinoembryonic antigen; EGFR, epidermal growth factor receptor; EOCRC, early‐onset colorectal cancer; IRI, irinotecan; MSI‐H, microsatellite instability‐high; MSS, microsatellite stable; OX, oxaliplatin; RFA, radiofrequency ablation; TACE, transarterial chemoembolization; TARE, transarterial radioembolization; UK, unknown; WT, wild type.

^a^ Sensitivity analyses were performed for the missing data. Pooled *p*‐values of multiple imputations in sensitivity analysis were presented.

Compared with the synchronous metastatic ≥50‐year group, synchronous metastatic EOCRC patients more frequently underwent urgent surgery (32.1% vs. 11.1%, *p* = .014) and had local treatments such as transarterial radioembolization, transarterial chemoembolization, and radiofrequency ablation (39.3% vs. 20.4%, *p* = .037). Diabetes and hypertension were more common in the ≥50‐year group (23.1% vs. 3.6%, *p* = .019, and 37% vs. 10.7%, *p* = .008, respectively) (Table 3).

**FIGURE 2:**
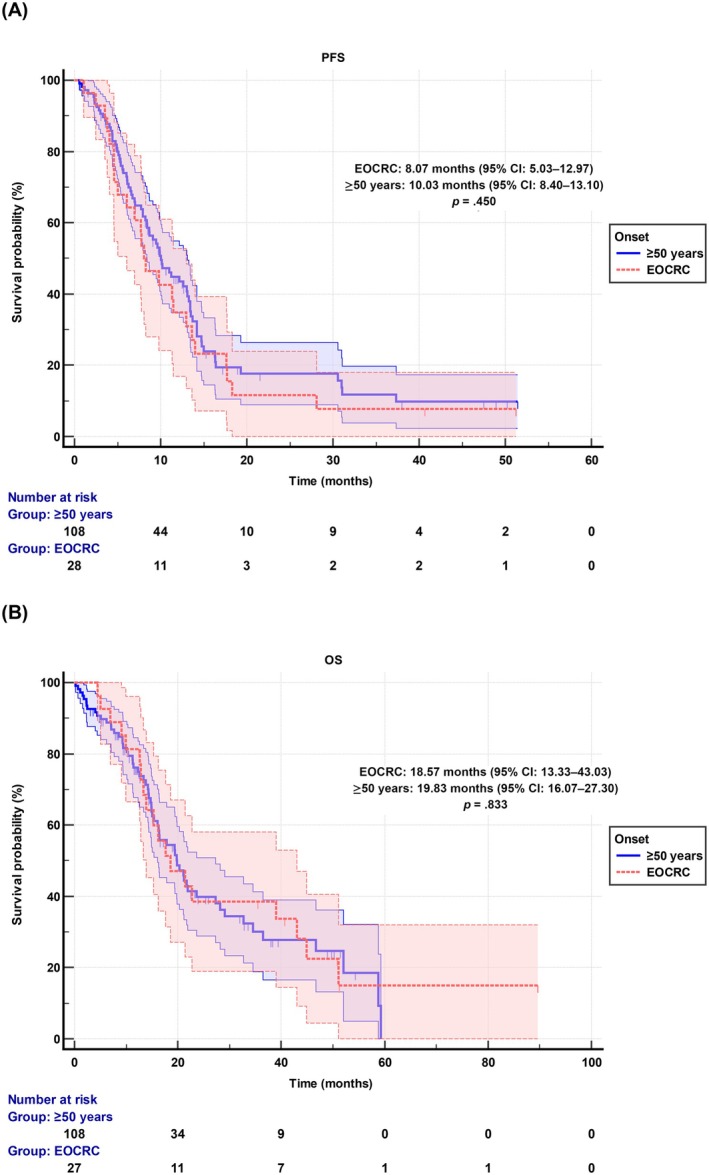
(A) Progression‐free survival and (B) overall survival (OS) of early‐onset colorectal cancer (EOCRC) and ≥50‐year‐old synchronous metastatic CRC patients.

Median PFS was 8.07 months (95% CI: 5.03–12.97) in the EOCRC group and 10.03 months (95% CI: 8.40–13.10) in the ≥50‐year group (*p* = .450) (Figure 2A). Median OS was 18.57 months (95% CI: 13.33–43.03) and 19.83 months (95% CI: 16.07–27.30), respectively (*p* = .833) (Figure 2B). Analyses of factors associated with PFS and OS in EOCRC patients revealed that only the location of the tumor (rectal vs. colon) was significantly associated with PFS (HR: 2.72 [95% CI: 1.15–6.45], *p* = .023) and OS (HR: 2.65 [95% CI: 1.02–6.86], *p* = .044) (Table S6).

### 3.3 Localized EOCRC and synchronous metastatic EOCRC

Applicable demographic and clinical characteristics were compared between localized EOCRC and synchronous metastatic EOCRC to investigate any parameters associated with disease presentation in EOCRC patients. 93.5% of localized and 96.5% of synchronous metastatic diseases were symptomatic at diagnosis. Abdominal pain (59.7% vs. 53.6%, *p* = .587), rectal bleeding (33.9% vs. 42.9%, *p* = .413), and constipation‐tenesmus (29.0% vs. 28.6%, *p* = .964) were most common presenting symptoms in both groups with no significant difference (Table 4). Age, gender, comorbidities, primary tumor location, MSI status, *BRAF* status, ABO, and Rh blood groups did not differ between localized and synchronous metastatic EOCRC patients (Table 4). However, synchronous metastatic EOCRC patients more frequently underwent urgent surgery (32.1% vs. 8%, *p* = .008) and more commonly had *RAS* mutation (43.5% vs. 16.7%, *p* = .032, *p* in sensitivity analysis = .001) (Table 4).

**TABLE 4:** Comparison of localized vs. synchronous metastatic early‐onset colorectal cancer (EOCRC) patients.

Variable	Localized EOCRC	Synchronous metastatic EOCRC	*p*‐Value
	*n* = 62	*n* = 28	
Age (median, min–max)	46 (20–49)	45 (30–49)	.470
Gender, *n* (%)			
Male	36 (58.1)	17 (60.7)	.813
Female	26 (41.9)	11 (39.3)	
Diabetes, *n* (%)	5 (8.1)	1 (3.6)	.661
Hypertension, *n* (%)	3 (4.8)	3 (10.7)	.370
CAD, *n* (%)	1 (1.6)	0 (0)	1.000
Symptomatic diagnosis, *n* (%)	58 (93.5)	27 (96.4)	.581
Presenting symptoms, *n* (%)			
Abdominal pain	37 (59.7)	15 (53.6)	.587
Rectal bleeding	21 (33.9)	12 (42.9)	.413
Constipation‐tenesmus	18 (29)	8 (28.6)	.964
Diarrhea	8 (12.9)	4 (14.3)	.858
Nausea‐vomiting	8 (12.9)	3 (10.7)	.769
Weight loss	7 (11.3)	6 (21.4)	.205
Location, *n* (%)			
Colon	42 (67.7)	15 (53.6)	.197
Rectum	20 (32.3)	13 (46.4)	
Urgent surgery, *n* (%)	5 (8)	9 (32.1)	.**008**
MSI, *n* (%)			
MSI‐H	9 (14.5)	2 (7.1)	.631/*1* ^a^
MSS	51 (82.3)	19 (67.9)	
UK	2 (3.2)	7 (25)	
*KRAS or NRAS*, *n* (%)			
WT	25 (40.3) (in‐known 83.3)	13 (46.4) (in‐known 56.5)	.**032/.001** ^a^
Mutant	5 (8) (in‐known 16.7)	10 (35.7) (in‐known 43.5)	
UK	32 (51.6)	5 (17.9)	
*BRAF V600E*, *n* (%)			
WT	30 (48.4)	20 (71.4)	.417/.*350* ^a^
Mutant	1 (1.6)	0 (0)	
UK	31 (50)	8 (28.6)	
ABO group, *n* (%)			
AB	6 (9.7)	2 (7.1)	.612/.*390* ^a^
A	31 (50)	10 (35.7)	
B	6 (9.7)	4 (14.4)	
O	17 (27.4)	10 (35.7)	
UK	2 (3.2)	2 (7.1)	
Rh, *n* (%)			
Positive	50 (80.6)	24 (85.8)	.332/.*480* ^a^
Negative	10 (16.1)	2 (7.1)	
UK	2 (3.2)	2 (7.1)	

*Note*: Statistically significant *p* values (based on a two‐tailed test of significance, *p* = .05) were written bold.

Abbreviations: CAD, coronary artery disease; CRC, colorectal cancer; In‐known, percentage among patients with known test result; MSI‐H, microsatellite instability‐high; UK, unknown; WT, wild type.

^a^ Sensitivity analyses were performed for the missing data. Pooled *p*‐values of multiple imputations in sensitivity analysis were presented.

## 4 DISCUSSION

In this study, we investigated demographic, clinical, treatment, and survival characteristics of localized and synchronous metastatic EOCRC patients, comparing them with the ≥50‐year group. We additionally investigated factors potentially associated with the synchronous metastatic presentation in EOCRC.

EOCRC percentage among all CRCs is reported from 0.4% to as high as 35.6% with a median of 7% in different studies.^10^ In our study, 14.4% of the localized and 20.6% of the synchronous metastatic cohort were EOCRC, comparable with the literature reflecting the increasing rate of EOCRC. It has been reported that EOCRC patients are more likely to be women.^2^ However, only localized EOCRC was associated with female dominance in our study, while synchronous metastatic EOCRC was not.

Most of the early‐onset gastrointestinal cancers are sporadic and lack a hereditary or familial background. High body mass index, high fasting plasma glucose, smoking, alcohol use, low physical activity, antibiotics, microbiome, and environmental exposures may have a role.^11^ Obesity, diabetes, and insulin resistance are associated with increased risk of CRC.^12^, ^13^ Moreover, obesity and metabolic syndrome may increase the risk of EOCRC.^4^, ^14^, ^15^ In our study, EOCRC patients in both localized and synchronous metastatic cohorts had lower rates of diabetes, hypertension, and CAD compared with ≥50‐year patients, probably due to increased prevalence of comorbidities by age. Yet, the very low rate of diabetes and hypertension in EOCRC patients in our study may suggest that a complete metabolic syndrome profile may not be necessary for the development of EOCRC. Obesity and poor diet quality may potentially contribute to carcinogenesis. In a systematic meta‐analysis including 32 studies of patients with EOCRC, the proportion of MSI‐H patients was found to be similar to all CRC patients (approximately 10%), and similar to our study; thus, MSI alone does not elucidate the pathogenesis of EOCRC.^16^ However, dramatic advancements have been made in treating MSI‐H CRC by immunotherapy in recent years.^17^ Neoadjuvant nivolumab‐ipilimumab in MSH‐H colon cancer provided a pathologic response rate of 98% (68% complete response).^18^ In first‐line treatment of MSI‐H metastatic CRC, pembrolizumab provided significantly longer OS compared with standard‐of‐care chemotherapy (not reached vs. 36.7 months, HR: 0.74, 95% CI 0.53–1.03; *p* = 0.036) and became the standard recommendation for the first line.^19^ These dramatic results underscore the critical need to address barriers that prevent these young EOCRC patients from accessing immunotherapy, which is still faced globally.

As an interesting finding, PNI was much more common in localized EOCRC patients compared with ≥50‐year patients (41.9% vs. 24.9%, *p* = .005). Previously, Hashmi et al.^20^ also reported that EOCRC showed a higher frequency of PNI (42.6% vs. 23.7%, *p* = .001). PNI may be recognized as pathological evidence of early metastasis, increasing incidence by stage, thereby associated with worse survival.^21^ The higher PNI incidence in localized EOCRC patients may reflect more aggressive tumor biology, different molecular mechanisms of carcinogenesis, and tumor microenvironment in EOCRC.

Among localized patients, the folinic acid, 5‐fluorouracil, and oxaliplatin chemotherapy regimen was more frequently preferred for EOCRC, while oxaliplatin, capecitabine, and capecitabine were more common among the ≥50‐year group. Moreover, perioperative chemotherapy cycles were significantly higher in the EOCRC group. Better performance scores, less comorbidity, and possible bias in the treatment of young patients may affect treatment intensity and duration. Despite several differences in clinical and treatment characteristics, the RFS and OS did not significantly differ between EOCRC and ≥50‐year groups. Although RFS is not statistically different in our study, the hazard plots revealed a tendency of worse survival in EOCRC patients. Hereby, a plausible interpretation may be that younger age, fewer comorbidities, and more intensive chemotherapy may compensate for disease aggressiveness in EOCRC patients.

Previous reports revealed that EOCRC patients are more symptomatic at the diagnosis, with the symptoms of rectal bleeding and abdominal pain.^22^ In our study, more than 90% of patients with localized and synchronous metastatic disease were symptomatic at diagnosis. The high rate of symptoms at diagnosis even in the early stages emphasizes the importance of screening for these young populations. Abdominal pain and rectal bleeding were the most common symptoms, consistent with previous reports. Differential diagnosis of these symptoms is crucial, and malignancy potential should not be neglected in young patients.

In our study, EOCRC patients needed urgent surgery more frequently than the ≥50‐year group among patients with synchronous metastases. However, no significant difference was observed among localized patients. A higher rate of urgent surgery among synchronous metastatic EOCRC patients may reflect a higher tumor burden at the diagnosis. However, local treatments such as transarterial radioembolization, transarterial chemoembolization, and radiofrequency ablation were also more common among EOCRC patients in the synchronous metastatic setting. The intention of a curative approach may be more common among young patients as a bias. Yet, PFS and OS were not different between EOCRC and ≥50‐year patients in this group. Interestingly, only localization of the primary tumor was associated with PFS and OS, indicating a worse prognosis for rectal tumors.

It has been shown that EOCRC patients are more likely diagnosed with advanced disease.^2^ The comparison of demographic and clinical parameters between localized and synchronous metastatic patients in our study, however, did not show differences except the need for urgent surgery and *RAS* mutant status. Synchronous metastatic EOCRC patients need urgent surgery more often than localized EOCRC as well as synchronous metastatic ≥50‐year patients. This phenomenon suggests that late diagnosis is an issue among EOCRC patients, emerging questions on screening younger individuals.^2^

*RAS* mutations occur early in CRC carcinogenesis and are maintained in the progression of the disease.^23^ Although *RAS* mutations have been reported to be related to worse prognosis in CRC, the data are controversial hindering definitive conclusions.^24^ It has been shown that *KRAS* mutations are associated with worse prognosis in young‐onset patients, especially in distal tumors.^25^ In our study, *RAS* mutant status was associated with shorter RFS in localized EOCRC patients. Moreover, synchronous metastatic EOCRC patients had *RAS* mutations more often than localized EOCRC patients. These data suggest that *RAS* mutation status may have a role in prognosis and presenting stage among EOCRC patients.

Advancements in treatment armamentarium of CRC, such as immunotherapy and *RAS* inhibitors (targeting *KRAS* G12C, panKRAS, and panRAS inhibitors), may affect the prognosis and management of EOCRC patients in the near future.^26^ The data on efficacy and safety of novel therapies in EOCRC patients are not defined yet, since adolescents and young adults are under‐represented populations in clinical trials.^27^ In melanoma, young patients had more frequent *BRAF* mutation, and complete response rates to immune checkpoint inhibitors and toxicity profiles differed from average‐aged patients.^28^ Long‐term adverse events such as endocrinological changes and fertility concerns require caution.^28^

There are several limitations of this study. Firstly, the study contains all the limitations of retrospective design. Missing data may decrease the quality of analyses for individual variables, despite sensitivity analyses. The study is a single‐center study, which limits the generalizability of the findings. External validation in a multicenter setting would enhance the study's external validity and confirm the reproducibility of the results. Therefore, a multicenter prospective study, which is being planned, would provide stronger data on EOCRC patients. Although our study is a single‐center study, this eliminates different approaches in patient management between centers, facilitating comparison between cohorts. The periods of the cohorts slightly differed between localized and synchronous metastatic groups, precluding the covering of the whole spectrum of EOCRC. Finally, novel treatments, such as immunotherapy in MSI‐H tumors, are not well‐established in our country. Further studies may show different characteristics of EOCRC patients in the era of novel treatments such as immunotherapy and *RAS* inhibitors.

## 5 CONCLUSION

In conclusion, localized and synchronous metastatic EOCRC patients may have several distinct demographic, clinical, and treatment characteristics. The prognosis of EOCRC patients did not differ from ≥50‐year patients, either in the localized or synchronous metastatic setting. *RAS* mutant status may be a prognostic factor in EOCRC. Implementation of stage‐specific characteristics of EOCRC into daily practice is necessary for decision‐making processes in the diagnosis and management of these young patients.

## AUTHOR CONTRIBUTIONS

**Erman Akkus:** Conceptualization; investigation; writing – original draft; methodology; writing – review and editing; data curation; formal analysis. **Beliz Bahar Karaoğlan:** Conceptualization; investigation; methodology; writing – review and editing; formal analysis; data curation. **Mehmet Kayaalp:** Writing – review and editing; formal analysis; methodology; investigation; data curation. **Utkucan Turmuş:** Investigation; writing – review and editing; methodology; data curation. **Cihangir Akyol:** Data curation; methodology; supervision; writing – review and editing; conceptualization; investigation. **Güngör Utkan:** Conceptualization; investigation; writing – review and editing; supervision; methodology.

## CONFLICT OF INTEREST STATEMENT

The authors have no relevant financial or non‐financial interests to disclose.

## ETHICS STATEMENT

Ethical approval was obtained from the Clinical Research Ethics Committee of Ankara University Faculty of Medicine (Number: 2022000659, 2022/659 and Number: 2023000367–1, 2023/367) in compliance with the Declaration of Helsinki. The study analyzed retrospective, anonymous clinical data of the patients. Therefore, informed consent of the patients was not required, and waiver/exemption was granted by the Ethics Committee for this purpose.

## Supporting information


**Table S1.** Multivariable binary logistic analysis for localized EOCRC*.
**Table S2.** Multivariable binary logistic analysis for localized EOCRC*.
**Table S3.** Univariable Cox regression analysis for recurrence‐free survival (RFS) in localized EOCRC patients.
**Table S4.** Multivariable Cox regression analysis for recurrence‐free survival (RFS) in localized EOCRC patients.
**Table S5.** Multivariable Cox regression analysis for recurrence‐free survival (RFS) in localized EOCRC patients (additionally adjusted by basic characteristics).
**Table S6.** Univariable Cox regression analyses for PFS and OS in synchronous metastatic EOCRC patients.

## Data Availability

The data that support the findings of this study are available on request from the corresponding author.
