# Supplementary material for: Stage‐specific characterization of “early‐onset colorectal cancer”: Localized and synchronous metastatic disease
Source: Int J Cancer. 2025 Jan 30;156(12):2340–51. doi: 10.1002/ijc.35336 (PMC12008821; doi:10.1002/ijc.35336)
Supplement: Supplementary file 1 — Table S1. Multivariable binary logistic analysis for localized EOCRC*. Table S2. Multivariable binary logistic analysis for localized EOCRC*. Table S3. Univariable Cox regression analysis for recurrence‐free survival (RFS) in localized EOCRC patients. Table S4. Multivariable Cox regression analysis for recurrence‐free survival (RFS) in localized EOCRC patients. Table S5. Multivariable Cox regression analysis for recurrence‐free survival (RFS) in localized EOCRC patients (additionally adjusted by basic characteristics). Table S6. Univariable Cox regression analyses for PFS and OS in synchronous metastatic EOCRC patients. [file IJC-156-2340-s001.pdf]

Supplementary

Stage-Specific Characterization of "Early-Onset Colorectal Cancer": Localized and Synchronous Metastatic Disease

Erman Akkus, Beliz Bahar Karaoğlan, Mehmet Kayaalp, Utkucan Turmuş, Cihangir Akyol, Güngör Utkan

Contents:

Supplementary tables

Table S1

Table S2

Table S3

Table S4

Table S5

Table S6

Supplementary Tables

Table S1. Multivariable binary logistic analysis for localized EOCRC*

**Variable**$^{+}$	**OR (95% CI)**	**P**
**Gender (Female vs male)**	2.17 (1.24-3.77)	**0.006**
**PNI (Yes vs No)**	2.28 (1.30-4.02)	**0.004**

*** a significant model (chi-square=14.950, df=2, p=0.001), constant p=0.000**

$^{+}$ **variables that were statistically significant in univariable analysis were included.**

Table S2. Multivariable binary logistic analysis for localized EOCRC *

**Variable**$^{+}$	**OR (95% CI)**	**P**
**Gender (Female vs male)**	2.26 (1.28-4.01)	**0.005**
**PNI (Yes vs No)**	2.15 (1.20-3.85)	**0.010**
**Diabetes (Yes vs No)**	0.64 (0.23-1.77)	0.393
**Hypertension (Yes vs No)**	0.17 (0.05-0.61)	**0.007**
**CAD (Yes vs No)**	0.27 (0.03-2.23)	0.229

*** a significant model (chi-square=39.940, df=5, p=0.000), constant p=0.001**

$^{+}$ **variables that were statistically significant in univariable analysis were included.**

Table S3. Univariable Cox regression analysis for recurrence-free survival (RFS) in localized EOCRC patients

Variable	HR (95% CI)	P
**Gender, (Male vs. female)**	1.07 (0.37-3.02)	0.896
**Diabetes (Present vs absent)**	0.04 (0.00-48.36)	0.377
**Hypertension, (Present vs absent)**	1.03 (0.13-7.89)	0.973
**CAD (Present vs absent)**	0.04 (0.00-864.81)	0.722
**Primary tumor location,**		
**Transverse vs right**	0.66 (0.07-5.97)	0.715
**Left vs right**	0.55 (0.13-2.24)	0.411
**Rectum vs right**	1.29 (0.36-4.60)	0.689
**Urgent surgery (Obstruction vs. no)**	2.57 (0.57-11.52)	0.517
**T stage**		
**3 vs 2**	NC	0.935
**4 vs 2**	NC	0.931
**N stage**		
**1a-b vs 0**	2.08 (0.56-7.78)	0.272
**1c vs 0**	3.60 (0.80-16.18)	**0.094**
**2 vs 0**	3.72 (0.82-16.729	**0.086**
**Tumor deposit (Present vs absent)**	1.34 (0.30-5.98)	0.695
**Tumor grade, n (%)**		
**2 vs 1**	0.71 (0.08-5.73)	0.752
	S: 0.79 (0.10-5.90)*	*0.825*
**3 vs 1**	1.64 (0.14-18.31)	0.687
	S:2.01 (0.20-19.49)	*0.545*
**Mucinous component (Present vs absent)**	1.14 (0.36-3.58)	0.822
**LVI (Present vs absent)**	1.62 (0.57-4.58)	0.358
**PNI (Present vs absent)**	1.16 (0.42-3.22)	0.769
**Budding (Present vs absent)**	1.24 (0.34-4.44)	0.735
**MSI-H (Yes vs No)**	1.19 (0.26-5.36)	0.814
	S: 1.28 (0.29-5.58)	*0.741*
**RAS (Mutant vs WT)**	**7.09 (1.87-26.76) /**	**<0.001**
	S: 15.06 (3.35-67.73)	***0.001***
**RAF, n (%)**		
**Mutant vs WT**	0.04 (0.00-3977)	0.592
	S: 2.60 (0.63-10.71)	*0.180*
**ABO group**		
**A vs AB**	0.91 (0.19-4.30)	0.906
	S: 0.91 (0.19-4.45)	*0.916*
**B vs AB**	NC	0.985
	S: 0.80 (0.07-9.23)	*0.862*
**O vs AB**	0.83 (0.15-4.55)	0.830
	S: 0.85 (0.15-4.77)	*0.856*
**Rh (Negative vs positive)**	0.24 (0.03-1.84)	0.170
	S: 0.22 (0.02-1.69)	*0.146*
**Perioperative treatment (Yes vs No)**	0.04 (0.00-55.20)	0.38
**Adjuvant chemotherapy,**		
**XELOX vs No**	NC	0.928
**FOLFOX vs No**	NC	0.925
**Capecitabine vs No**	NC	1
**Neoadjuvant radiotherapy**		
**Short course (5x5) vs No**	1.60 (0.20-12.78)	0.654
**Long Course (CRT) vs No**	2.38 (0.79-7.17)	**0.122**
**Neoadjuvant chemotherapy**		
**XELOX vs No**	NC	0.438
**FOLFOX vs No**	NC	0.737

Abbreviations: EOCRC: early-onset colorectal cancer, CRC: colorectal cancer, CAD: coronary artery disease, LVI: lymphovascular invasion, PNI: perineural invasion, MSI-H: microsatellite instability-high, WT: wild type, NC: not calculated, CRT: chemoradiotherapy, FOLFOX: Folinic acid, 5-fluorouracil and oxaliplatin, XELOX: Oxaliplatin and capecitabine [S: sensitivity analysis:* Sensitivity analyses were performed for the variables that had missing data. Pooled results of multiple imputations were presented.]**

Table S4. Multivariable Cox regression analysis for recurrence-free survival (RFS) in localized EOCRC patients

Variable	HR (95% CI)	P
**N stage**		
**1a-b vs 0**	1.62 (0.27-9.52)	0.59
	S: 1.90 (0.45-7.94)	*0.377*
**1c vs 0**	**51.92 (4.36-618.08)**	**0.002**
	S: 3.32 (0.68-16.18)	*0.136*
**2 vs 0**	2.54 (0.30-20.95)	0.38
	S: 4.47 (0.93-21.46)	*0.061*
**RAS (Mutant vs WT)**	**14.25 (2.61-77.61)**	**0.002**
	S: 14.83 (3.00-73.15)	***0.001***
**Neoadjuvant radiotherapy**		
**Short course (5x5) vs No**	NC	
	S: 1.06 (0.08-12.60)	*S: 0.963*
**Long Course (CRT) vs No**	2.39 (0.50-11.33)	0.27
	S: 1.78 (0.52-6.05)	*0.351*

Abbreviations: EOCRC: early-onset colorectal cancer, WT: wild type, NC: not calculated, CRT: chemoradiotherapy, [S: sensitivity analysis:* Sensitivity analyses were performed for the variables that had missing data. Pooled results of multiple imputations were presented.]**

Table S5. Multivariable Cox regression analysis for recurrence-free survival (RFS) in localized EOCRC patients (additionally adjusted by basic characteristics)

Variable	HR (95% CI)	P
**Age (continuous variable)**	0.95 (0.87-1.04)	0.321
**Gender, (Male vs. female)**	0.29 (0.02-4.31)	0.372
**Hypertension, (Present vs absent)**	37.70 (0.89-145.68)	0.059
**Diabetes (Present vs absent)**	0.00 (0.00-NC)	0.866
**CAD (Present vs absent)**	0.00 (0.00-NC)	0.975
**Primary tumor location**		
**Transverse vs right**	NC	NC
**Left vs right**	1.40 (0.03-53.87)	0.855
**Rectum vs right**	0.02 (0.00-NC)	0.962
**N stage**		
**1a-b vs 0**	3.70 (0.26-52.47)	0.333
**1c vs 0**	**249.55 (3.11-2000.18)**	**0.014**
**2 vs 0**	6.58 (0.15-272.11)	0.321
**RAS (Mutant vs WT)**	**32.92 (2.32-467.05)**	**0.010**
**Neoadjuvant radiotherapy**		
**Short course (5x5) vs No**	NC	0.980
**Long Course (CRT) vs No**	NC	0.946

Abbreviations: EOCRC: early-onset colorectal cancer, CAD: coronary artery disease WT: wild type, NC: not calculated, CRT: chemoradiotherapy

Table S6. Univariable Cox regression analyses for PFS and OS in synchronous metastatic EOCRC patients

	PFS		OS	
**Variable**	**OR (95% CI)**	**P**	**OR (95% CI)**	**P**
**Gender (Female vs male)**	0.77 (0.34-1.76)	0.542	1.16 (0.45-3.00)	0.748
**Diabetes (Absent vs present)**	0.91 (0.12-6.93)	0.932	25.94 (0.02-277.87)	0.360
**Hypertension (Absent vs present)**	1.12 (0.33-3.82)	0.851	2.36 (0.50-11.02)	0.272
**CAD (Absent vs present)**	NC	NC	NC	NC
**Smoking history (Absent vs present)**	1.12 (0.54-2.76)	0.623	1.32 (0.51-3.41)	0.554
**Location (Rectum vs colon)**	**2.72 (1.15-6.45)**	**0.023**	**2.65 (1.02-6.86)**	**0.044**
**MSI (High vs. stable)**	0.44 (0.05-3.43)<br>*S: 0.38 (0.07-1.94)*	0.436<br>*0.247*	0.58 (0.07-4.58)<br>*S: 0.31 (0.04-2.06)*	0.611<br>*0.223*
***RAS* (Mutant vs wild type)**	1.07 (0.43-2.61)<br>*S: 1.02 (0.42-2.42)*	0.879<br>*0.963*	0.94 (0.32-2.73)<br>*S: 0.80 (0.29-2.20)*	0.920<br>*0.672*
***RAF* (Mutant vs wild type)**	NC	NC	NC	NC
**Liver metastasis (Absent vs present)**	3.29 (0.69-15.60)	0.133	2.85 (0.60-13.52)	0.186
**Peritoneal metastasis (Absent vs present)**	0.73 (0.09-5.57)	0.763	0.16 (0.01-1.51)	0.112
**Lung metastasis (Absent vs present)**	0.54 (0.26-1.28)	0.164	0.47 (0.18-1.24)	0.130
**Bone metastasis (Absent vs present)**	NC	NC	NC	NC
**CEA (continuous variable)**	1.001 (1.000-1.002)	0.056	1.001 (1.000-1.002)	0.079
**Ca19-9 (continuous variable)**	1.00 (1.00-1.00)	0.344	1.00 (1.00-1.00)	0.531
**Urgent surgery (Absent vs present)**	1.94 (0.80-4.74)	0.141	3.39 (0.97-11.79)	0.055
**First-line treatment (all treatments compared to 5FU-OX doublet)**	All 1.000 with varying 95% CI	All p is 1.000	All 1.000 with varying 95% CI	All p is 1.000
**Local treatment (Absent vs present)**	0.99 (0.44-2.25)	0.997	1.71 (0.68-4.31)	0.250
**Surgery as a local treatment (Absent vs present)**	1.19 (0.27-5.26)	0.811	29.87 (0.16-545,359	0.201
**TARE-TACE-RF as local treatments (Absent vs present)**	1.05 (0.46-2.40)	0.895	1.26 (0.50-3.16)	0.617
**ABO**				
**A vs AB**	0.32 (0.06-1.64)<br>*S: 0.29 (0.06-1.45)*	0.176<br>*0.133*	0.44 (0.08-2.35)<br>*S: 0.68 (0.08-5.61)*	0.339<br>*0.717*
**B vs AB**	0.20 (0.03-1.36)<br>*S: 0.22 (0.03-1.43)*	0.102<br>*0.115*	0.48 (0.07-3.21)<br>*S: 0.78 (0.08-7.06)*	0.449<br>*0.828*
**0 vs AB**	0.36 (0.07-1.84)<br>*S: 0.36 (0.047-1.80)*	0.225<br>*0.217*	0.87 (0.17-4.29)<br>*S: 1.14 (0.17-7.51)*	0.872<br>*0.885*
**Rh (negative vs positive)**	1.04 (0.24-4.54)<br>*S: 1.12 (0.26-4.85)*	0.951<br>*0.876*	0.79 (0.10-6.10)<br>*S: 0.53 (0.05-5.36)*	0.825<br>*0.580*

EOCRC: Early onset colorectal cancer, CRC: colorectal cancer, CAD: coronary artery disease, LVI: lymphovascular invasion, PNI: perineural invasion, MSI-H: microsatellite instability-high, WT: wild type, UK: unknown, 5FU: 5-fluorouracil, OX: oxaliplatin, IRI: irinotecan, EGFR: epidermal growth factor receptor, TARE: Transarterial radioembolization, TACE: Transarterial chemoembolization, RFA: Radiofrequency ablation, NC: Not calculated.
[S: sensitivity analysis:* Sensitivity analyses were performed for the variables that had missing data. Pooled results of multiple imputations were presented.]**
